# *cis*-3,3-Dimethyl-3,3a,4,5,6,6a-hexa­hydro-1*H*-cyclo­penta­[*c*]furan-1,6-dione

**DOI:** 10.1107/S1600536808017133

**Published:** 2008-06-13

**Authors:** Wayne H. Pearson, Stacey E. Lanham, Debra K. Dillner

**Affiliations:** a Chemistry Department, United States Naval Academy, 572M Holloway Road, Annapolis, Maryland 21402, USA

## Abstract

The bicyclic mol­ecule of the title compound, C_9_H_12_O_3_, contains two five-membered rings with different functional groups, *viz*. a ketone and an ester. Both rings assume an envelope conformation. The mean planes of these functional groups form a dihedral angle of 60.7 (1)°. The crystal structure exhibits weak inter­molecular C—H⋯O inter­actions, which link the mol­ecules into zigzag chains extended in the [010] direction. The unit cell contains a racemic mixture of enanti­omers.

## Related literature

For related literature, see: Boeckman *et al.* (1989 ▶); Wang *et al.* (2006 ▶); Rodriguez (1998 ▶); Corey & Kang (1984 ▶).
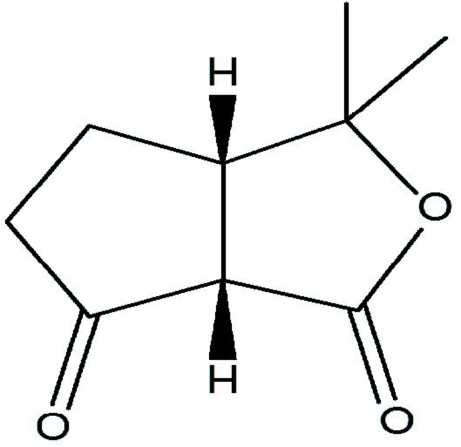


## Experimental

### 

#### Crystal data

C_9_H_12_O_3_
*M* *_r_* = 168.19
Triclinic, 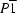

*a* = 6.7333 (7) Å
*b* = 8.2897 (8) Å
*c* = 8.5906 (8) Å
α = 111.657 (2)°
β = 103.571 (2)°
γ = 92.809 (2)°
*V* = 428.30 (7) Å^3^
*Z* = 2
Mo *K*α radiation
μ = 0.10 mm^−1^
*T* = 173 (2) K
0.33 × 0.16 × 0.13 mm

#### Data collection

Bruker Kappa APEXII diffractometer
Absorption correction: multi-scan (*SADABS*; Bruker, 2007 ▶) *T*_min_ = 0.934, *T*_max_ = 0.988
9157 measured reflections
1961 independent reflections
1632 reflections with *I* > 2σ(*I*)
*R*_int_ = 0.067

#### Refinement

*R*[*F* ^2^ > 2σ(*F* ^2^)] = 0.038
*wR*(*F* ^2^) = 0.107
*S* = 1.06
1961 reflections
111 parameters
H-atom parameters constrained
Δρ_max_ = 0.34 e Å^−3^
Δρ_min_ = −0.16 e Å^−3^

### 

Data collection: *APEX2* (Bruker, 2007 ▶); cell refinement: *APEX2*; data reduction: *APEX2*; program(s) used to solve structure: *SHELXTL* (Sheldrick, 2008 ▶); program(s) used to refine structure: *SHELXTL*; molecular graphics: *SHELXTL*; software used to prepare material for publication: *SHELXTL*.

## Supplementary Material

Crystal structure: contains datablocks I, global. DOI: 10.1107/S1600536808017133/cv2418sup1.cif

Structure factors: contains datablocks I. DOI: 10.1107/S1600536808017133/cv2418Isup2.hkl

Additional supplementary materials:  crystallographic information; 3D view; checkCIF report

## Figures and Tables

**Table 1:** Hydrogen-bond geometry (Å, °)

*D*—H⋯*A*	*D*—H	H⋯*A*	*D*⋯*A*	*D*—H⋯*A*
C6—H6⋯O3^i^	1.00	2.51	3.3418 (13)	140
C4—H4*B*⋯O2^ii^	0.99	2.51	3.4821 (16)	166

Symmetry codes: (i) 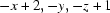; (ii) 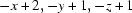.

**Fig. 1.**
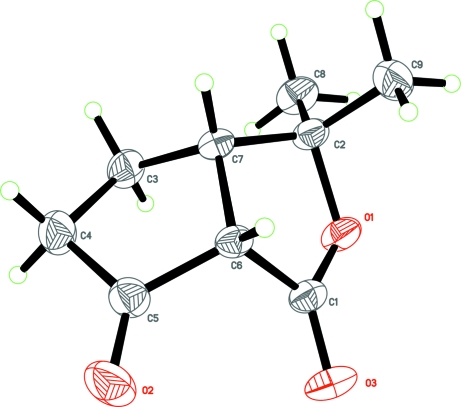
The molecular structure of the title compound, with atom labels and 50% probability displacement ellipsoids for non-H atoms.

## supplementary crystallographic information

### Comment

Cyclopentane rings bearing multiple stereocenters are a common motif in terpenes, including ceroplastol (Boeckman *et al.*, 1989) and dolabellanes. (Wang *et al.*, 2006) Due to the wide variety of biological activities shown by these terpenes, interest in their synthesis is high. (Rodriguez, 1998) The bicyclic nature of the title compound makes it conformationally rigid. This rigidity is essential to its use as a stereochemical control element. Subsequent transformations require that one face of the molecule be more accessible than the other. For this reason, obtaining a crystal structure was an important goal.

The more accessible face of the molecule is oriented on the top side of figure 1. The angle between least-squares planes defined by the ketone and ester functional groups is 60.7 (1) degrees. Bond distances in this compound are quite reasonable when compared with expected values. The carbon-carbon bonds average 1.528 (10) Å in length. The two carbonyl bonds have an average length of 1.206 (2) Å while the ring oxygen, O1, is positioned 1.344 (1) Å from C1 and 1.488 (1) Å from C2. Strain in the ring system is observed in the bond angles on opposite sides of the molecule. Angles on the carbonyl side of the compound are considerably less than the expected 120 ° for an *sp^2^* hybridized carbon. The angle defined by C4—C5—C6 is 107.93 (9) ° while the O1—C1—C6 angle is 110.06 (8) °. On the opposite side of the ring system, the C3—C7—C2 angle is more open at 117.00 (9) ° rather than the 109.5 ° that would expected around an *sp^3^* hybridized, central atom.

### Experimental

As part of a synthetic effort to prepare natural products, the title compound was prepared in a manner similar to that described by Corey & Kang (1984). Crystals were obtained by evaporation from ethanol.

### Refinement

Although all of the H atoms were located in difference Fourier maps, H-atoms were placed and then constrained to be at idealized positions. Methyl H atoms were positioned at 0.98 Å, methylene H atoms at 0.99 Å, and methyne H atoms at 1.00 Å from parent carbon atoms. A riding model was used during refinement. Methyl H atoms were allowed to rotate around the adjacent carbon-carbon bond with *U*_iso_(H) = 1.5 times *U*_eq_(C). Methylene H atoms were treated as idealized secondary H atoms with *U*_iso_(H) = 1.2 times *U*_eq_(C). Methyne H atoms were treated as idealized tertiary H atoms with *U*_iso_(H) = 1.2 times *U*_eq_(C).

### Crystal data

**Table:**

C_9_H_12_O_3_	*Z* = 2
*M**_r_* = 168.19	*F*_000_ = 180
Triclinic, *P*1	*D*_x_ = 1.304 Mg m^−^^3^*D*_m_ = 1.258 Mg m^−^^3^*D*_m_ measured by flotation
Hall symbol: -P 1	Melting point = 355–357 K
*a* = 6.7333 (7) Å	Mo *K*α radiation λ = 0.71073 Å
*b* = 8.2897 (8) Å	Cell parameters from 4358 reflections
*c* = 8.5906 (8) Å	θ = 2.7–27.5º
α = 111.657 (2)º	µ = 0.10 mm^−^^1^
β = 103.571 (2)º	*T* = 173 (2) K
γ = 92.809 (2)º	Regular parallelepiped, colourless
*V* = 428.30 (7) Å^3^	0.33 × 0.16 × 0.13 mm

### Data collection

**Table:**

Bruker KAPPA APEXII diffractometer	1961 independent reflections
Radiation source: fine-focus sealed tube	1632 reflections with *I* > 2σ(*I*)
Monochromator: graphite	*R*_int_ = 0.067
Detector resolution: 512 pixels mm^-1^	θ_max_ = 27.5º
*T* = 173(2) K	θ_min_ = 2.7º
combination of ω and φ scans	*h* = −8→8
Absorption correction: multi-scan(SADABS; Bruker, 2007)	*k* = −10→10
*T*_min_ = 0.934, *T*_max_ = 0.988	*l* = −11→11
9157 measured reflections	

### Refinement

**Table:**

Refinement on *F*^2^	Secondary atom site location: difference Fourier map
Least-squares matrix: full	Hydrogen site location: inferred from neighbouring sites
*R*[*F*^2^ > 2σ(*F*^2^)] = 0.038	H-atom parameters constrained
*wR*(*F*^2^) = 0.108	*w* = 1/[σ^2^(*F*_o_^2^) + (0.057*P*)^2^ + 0.0541*P*] where *P* = (*F*_o_^2^ + 2*F*_c_^2^)/3
*S* = 1.06	(Δ/σ)_max_ < 0.001
1961 reflections	Δρ_max_ = 0.34 e Å^−^^3^
111 parameters	Δρ_min_ = −0.16 e Å^−^^3^
Primary atom site location: structure-invariant direct methods	Extinction correction: none

### Special details

**Table:**

Geometry. All e.s.d.'s (except the e.s.d. in the dihedral angle between two l.s. planes) are estimated using the full covariance matrix. The cell e.s.d.'s are taken into account individually in the estimation of e.s.d.'s in distances, angles and torsion angles; correlations between e.s.d.'s in cell parameters are only used when they are defined by crystal symmetry. An approximate (isotropic) treatment of cell e.s.d.'s is used for estimating e.s.d.'s involving l.s. planes.
Refinement. Refinement of *F*^2^ against ALL reflections. The weighted *R*-factor *wR* and goodness of fit *S* are based on *F*^2^, conventional *R*-factors *R* are based on *F*, with *F* set to zero for negative *F*^2^. The threshold expression of *F*^2^ > σ(*F*^2^) is used only for calculating *R*-factors(gt) *etc*. and is not relevant to the choice of reflections for refinement. *R*-factors based on *F*^2^ are statistically about twice as large as those based on *F*, and *R*- factors based on ALL data will be even larger.

### Fractional atomic coordinates and isotropic or equivalent isotropic displacement parameters (Å^2^)

**Table:**

	*x*	*y*	*z*	*U*_iso_*/*U*_eq_	
C1	1.03889 (16)	0.18603 (13)	0.76894 (14)	0.0248 (3)	
C2	0.75616 (16)	0.19250 (14)	0.88370 (14)	0.0252 (3)	
C3	0.67417 (18)	0.39922 (14)	0.71460 (15)	0.0295 (3)	
H3A	0.5373	0.4352	0.7209	0.035*	
H3B	0.7814	0.4840	0.8164	0.035*	
C4	0.7176 (2)	0.38907 (16)	0.54419 (17)	0.0347 (3)	
H4A	0.5888	0.3489	0.4478	0.042*	
H4B	0.7811	0.5049	0.5563	0.042*	
C5	0.86639 (18)	0.25662 (15)	0.51197 (15)	0.0291 (3)	
C6	0.84742 (15)	0.14672 (13)	0.61779 (13)	0.0219 (2)	
H6	0.8134	0.0185	0.5424	0.026*	
C7	0.67906 (15)	0.21095 (13)	0.70880 (13)	0.0218 (2)	
H7	0.5426	0.1354	0.6403	0.026*	
C8	0.70323 (19)	0.32707 (17)	1.03822 (15)	0.0340 (3)	
H8A	0.7730	0.3122	1.1447	0.051*	
H8B	0.5534	0.3105	1.0213	0.051*	
H8C	0.7491	0.4455	1.0484	0.051*	
C9	0.6960 (2)	0.00625 (16)	0.86661 (18)	0.0367 (3)	
H9A	0.7418	−0.0767	0.7715	0.055*	
H9B	0.5455	−0.0191	0.8417	0.055*	
H9C	0.7619	−0.0054	0.9757	0.055*	
O1	0.98475 (11)	0.22286 (11)	0.91791 (10)	0.0297 (2)	
O2	0.98035 (16)	0.23863 (12)	0.41910 (13)	0.0442 (3)	
O3	1.21632 (12)	0.18858 (11)	0.76418 (12)	0.0374 (2)	

### Atomic displacement parameters (Å^2^)

**Table:**

	*U*^11^	*U*^22^	*U*^33^	*U*^12^	*U*^13^	*U*^23^
C1	0.0204 (5)	0.0238 (5)	0.0290 (6)	0.0052 (4)	0.0081 (4)	0.0081 (4)
C2	0.0191 (5)	0.0320 (6)	0.0262 (6)	0.0068 (4)	0.0083 (4)	0.0115 (4)
C3	0.0307 (6)	0.0276 (6)	0.0334 (6)	0.0132 (4)	0.0132 (5)	0.0117 (5)
C4	0.0396 (7)	0.0349 (6)	0.0399 (7)	0.0161 (5)	0.0172 (6)	0.0211 (5)
C5	0.0317 (6)	0.0280 (5)	0.0307 (6)	0.0071 (4)	0.0131 (5)	0.0121 (5)
C6	0.0199 (5)	0.0204 (5)	0.0250 (5)	0.0046 (4)	0.0088 (4)	0.0067 (4)
C7	0.0174 (5)	0.0238 (5)	0.0228 (5)	0.0043 (4)	0.0066 (4)	0.0065 (4)
C8	0.0314 (6)	0.0449 (7)	0.0243 (6)	0.0107 (5)	0.0112 (5)	0.0091 (5)
C9	0.0382 (7)	0.0392 (7)	0.0457 (8)	0.0112 (5)	0.0213 (6)	0.0246 (6)
O1	0.0198 (4)	0.0411 (5)	0.0271 (4)	0.0078 (3)	0.0051 (3)	0.0124 (4)
O2	0.0564 (6)	0.0452 (5)	0.0526 (6)	0.0199 (4)	0.0381 (5)	0.0274 (5)
O3	0.0182 (4)	0.0444 (5)	0.0440 (5)	0.0058 (3)	0.0102 (4)	0.0097 (4)

### Geometric parameters (Å, °)

**Table:**

C1—O3	1.2043 (13)	C4—H4B	0.9900
C1—O1	1.3437 (13)	C5—O2	1.2076 (14)
C1—C6	1.5216 (15)	C5—C6	1.5255 (16)
C2—O1	1.4879 (12)	C6—C7	1.5322 (13)
C2—C8	1.5171 (15)	C6—H6	1.0000
C2—C9	1.5208 (16)	C7—H7	1.0000
C2—C7	1.5367 (16)	C8—H8A	0.9800
C3—C4	1.5325 (17)	C8—H8B	0.9800
C3—C7	1.5452 (15)	C8—H8C	0.9800
C3—H3A	0.9900	C9—H9A	0.9800
C3—H3B	0.9900	C9—H9B	0.9800
C4—C5	1.5162 (16)	C9—H9C	0.9800
C4—H4A	0.9900		
			
O3—C1—O1	122.38 (11)	C1—C6—C7	103.11 (8)
O3—C1—C6	127.55 (11)	C5—C6—C7	106.73 (8)
O1—C1—C6	110.06 (8)	C1—C6—H6	111.5
O1—C2—C8	106.91 (9)	C5—C6—H6	111.5
O1—C2—C9	107.27 (9)	C7—C6—H6	111.5
C8—C2—C9	111.54 (10)	C6—C7—C2	103.16 (8)
O1—C2—C7	102.78 (8)	C6—C7—C3	103.53 (8)
C8—C2—C7	116.17 (9)	C2—C7—C3	117.00 (9)
C9—C2—C7	111.33 (9)	C6—C7—H7	110.8
C4—C3—C7	104.52 (9)	C2—C7—H7	110.8
C4—C3—H3A	110.8	C3—C7—H7	110.8
C7—C3—H3A	110.8	C2—C8—H8A	109.5
C4—C3—H3B	110.8	C2—C8—H8B	109.5
C7—C3—H3B	110.8	H8A—C8—H8B	109.5
H3A—C3—H3B	108.9	C2—C8—H8C	109.5
C5—C4—C3	104.25 (9)	H8A—C8—H8C	109.5
C5—C4—H4A	110.9	H8B—C8—H8C	109.5
C3—C4—H4A	110.9	C2—C9—H9A	109.5
C5—C4—H4B	110.9	C2—C9—H9B	109.5
C3—C4—H4B	110.9	H9A—C9—H9B	109.5
H4A—C4—H4B	108.9	C2—C9—H9C	109.5
O2—C5—C4	126.95 (11)	H9A—C9—H9C	109.5
O2—C5—C6	125.12 (10)	H9B—C9—H9C	109.5
C4—C5—C6	107.93 (9)	C1—O1—C2	110.98 (8)
C1—C6—C5	112.00 (9)		
			
C7—C3—C4—C5	−34.23 (12)	O1—C2—C7—C6	30.96 (9)
C3—C4—C5—O2	−160.31 (13)	C8—C2—C7—C6	147.30 (9)
C3—C4—C5—C6	19.95 (13)	C9—C2—C7—C6	−83.58 (10)
O3—C1—C6—C5	−50.89 (15)	O1—C2—C7—C3	−81.93 (10)
O1—C1—C6—C5	127.96 (9)	C8—C2—C7—C3	34.42 (13)
O3—C1—C6—C7	−165.27 (11)	C9—C2—C7—C3	163.54 (9)
O1—C1—C6—C7	13.58 (11)	C4—C3—C7—C6	35.35 (11)
O2—C5—C6—C1	70.16 (15)	C4—C3—C7—C2	148.02 (9)
C4—C5—C6—C1	−110.10 (10)	O3—C1—O1—C2	−174.31 (10)
O2—C5—C6—C7	−177.71 (11)	C6—C1—O1—C2	6.77 (11)
C4—C5—C6—C7	2.03 (12)	C8—C2—O1—C1	−146.96 (10)
C1—C6—C7—C2	−27.21 (10)	C9—C2—O1—C1	93.29 (11)
C5—C6—C7—C2	−145.34 (9)	C7—C2—O1—C1	−24.16 (11)
C1—C6—C7—C3	95.19 (9)	H6—C6—C7—H7	−26.1
C5—C6—C7—C3	−22.94 (11)		

### Hydrogen-bond geometry (Å, °)

**Table:**

*D*—H···*A*	*D*—H	H···*A*	*D*···*A*	*D*—H···*A*
C6—H6···O3^i^	1.00	2.51	3.3418 (13)	140
C4—H4B···O2^ii^	0.99	2.51	3.4821 (16)	166

Symmetry codes: (i) −*x*+2, −*y*, −*z*+1; (ii) −*x*+2, −*y*+1, −*z*+1.
